# *In Vivo* Analysis of Conserved *C. elegans* Tomosyn Domains

**DOI:** 10.1371/journal.pone.0026185

**Published:** 2011-10-14

**Authors:** Anna O. Burdina, Susan M. Klosterman, Ludmila Shtessel, Shawn Ahmed, Janet E. Richmond

**Affiliations:** 1 Department of Biological Sciences, University of Illinois at Chicago, Chicago, Illinois, United States of America; 2 Department of Biology, The University of North Carolina at Chapel Hill, Chapel Hill, North Carolina, United States of America; Columbia University, United States of America

## Abstract

Neurosecretion is critically dependent on the assembly of a macromolecular complex between the SNARE proteins syntaxin, SNAP-25 and synaptobrevin. Evidence indicates that the binding of tomosyn to syntaxin and SNAP-25 interferes with this assembly, thereby negatively regulating both synaptic transmission and peptide release. Tomosyn has two conserved domains: an N-terminal encompassing multiple WD40 repeats predicted to form two β-propeller structures and a C-terminal SNARE-binding motif. To assess the function of each domain, we performed an *in vivo* analysis of the N- and C- terminal domains of *C. elegans* tomosyn (TOM-1) in a *tom-1* mutant background. We verified that both truncated TOM-1 constructs were transcribed at levels comparable to rescuing full-length TOM-1, were of the predicted size, and localized to synapses. Unlike full-length TOM-1, expression of the N- or C-terminal domains alone was unable to restore inhibitory control of synaptic transmission in *tom-1* mutants. Similarly, co-expression of both domains failed to restore TOM-1 function. In addition, neither the N- nor C-terminal domain inhibited release when expressed in a wild-type background. Based on these results, we conclude that the ability of tomosyn to regulate neurotransmitter release *in vivo* depends on the physical integrity of the protein, indicating that both N- and C-terminal domains are necessary but not sufficient for effective inhibition of release *in vivo*.

## Introduction

Synaptic vesicles undergo a priming process in which they become competent to fuse in response to a calcium signal [1]. During priming, the vesicle-associated SNARE synaptobrevin forms a stable complex with the plasma membrane SNAREs, SNAP-25 and syntaxin, bringing the vesicle in close apposition with the plasma membrane [2], [3], [4]. Several SNARE-interacting proteins have been shown to regulate priming, including the syntaxin-binding partner tomosyn, which acts as a negative regulator [5]. This conclusion is based on the inhibitory effects of tomosyn over-expression on release in several cell types [5], [6], [7], [8] and on enhanced synaptic transmission in both *C. elegans* and mouse mutants [9], [10], [11], [12]. However, the molecular events by which tomosyn mediates this inhibition remain to be fully elucidated.

Tomosyn has two conserved domains, a large N-terminal containing WD-40 repeats and a small C-terminal motif similar to the SNARE-binding domain of synaptobrevin [13], [14], [15]. In biochemical assays, the tomosyn SNARE domain can substitute for synaptobrevin, forming a 4-alpha helical bundle with syntaxin and SNAP-25, which closely resembles the crystal structure of the fusogenic SNARE complex [5], [6], [16]. Based on these observations, inhibition by tomosyn is thought to involve assembly of non-fusogenic tomosyn SNARE complexes at the expense of fusogenic SNARE complexes. However, while full-length tomosyn inhibits secretion in all cell types examined, expression of the C-terminal SNARE domain alone has produced variable results. For example, expression of the SNARE domain in chromaffin cells had no effect on the primed vesicle pool, and actually enhanced sustained release [17], whereas in cultured neurons and semi-intact PC12 cells, this domain produced partial inhibition [6], [12]. These data suggest that additional tomosyn domains may contribute to its inhibitory function. Consistent with this notion, tomosyn lacking a SNARE motif promotes SNARE complex oligomerization *in vitro* and inhibits secretion from chromaffin cells and superior cervical ganglion (SCG) neurons [8], [12], [17], [18]. Similarly, tomosyn with mutations in the SNARE domain that impair syntaxin binding, retains inhibitory function in PC12 secretion assays [19]. Brain extracts from mouse tomosyn mutants exhibit reduced levels of SNARE complex oligomers. Together, these observations suggest the tomosyn N terminus contributes to the regulation of secretion, possibly by limiting the availability of monomeric SNARE proteins. The tomosyn N terminus has also been shown to bind and inhibit synaptotagmin [20]. Thus, the current literature implicates both tomosyn domains in the regulation of secretion via several distinct molecular mechanisms. However, these roles have only been assayed *in cellulo*, and their interpretation is compounded by the presence of endogenous tomosyn. Here, we analyzed the independent and combined functionality of tomosyn N- and C-terminal domains at the *C. elegans* neuromuscular junction (NMJ) in a tomosyn mutant background.

## Results

### The relationship between TOM-1A expression levels and inhibitory synaptic function

**Figure 1:**
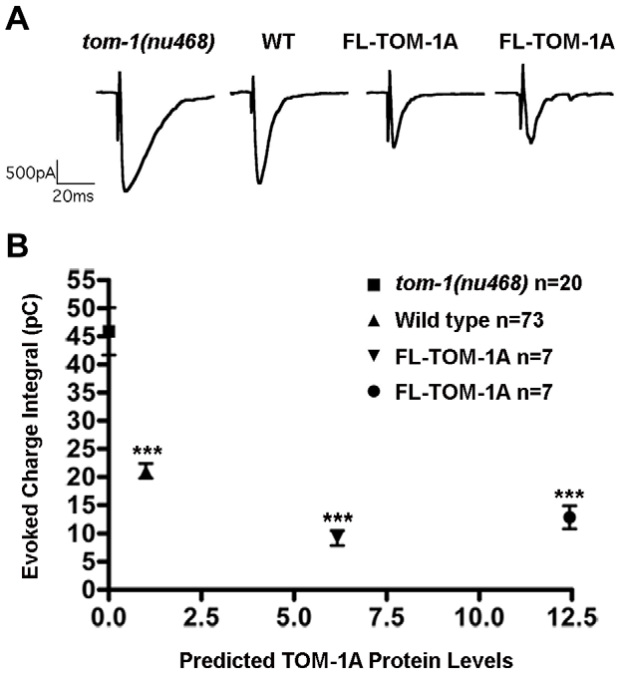
Inverse-relationship between predicted full-length TOM-1A expression levels and synaptic function. **A.** Representative evoked post-synaptic responses from the NMJ of *tom-1(nu468)*, wild type and two TOM-1A integrated lines, SY1229 and SY1242, expressed in the *tom-1(nu468)* mutant background respectively. **B.** Average charge integral for evoked responses of *tom-1(nu468)* (n = 20), wild type (n = 73) and *tom-1(nu468)* over-expressing TOM-1A integrated lines SY1242 (∼6 fold mRNA levels) (n = 7) and SY1229 (∼12 fold mRNA levels) (n = 7) plotted against predicted TOM-1A expression levels based on quantitative real-time RT-PCR (qRT-PCR) normalized to *C. elegans* actin (*act-1*) transcript levels. Data plotted as mean ± SEM (significance values relative to *tom-1(nu468)*, *** p≤0.0001, Mann Whitney T-test). Representative evoked NMJ traces are displayed above each strain.

*C. elegans tom-1* mutants exhibit an enhanced NMJ evoked response duration, resulting in an increased charge integral that reflects increased priming [10]. Re-introduction of full-length TOM-1A in cholinergic motor neurons reverses the *tom-1* mutant phenotype, producing inhibition of transmission relative to the wild type [10], [11]. This inhibitory effect was due to over-expression of TOM-1, a consequence of the standard method used to create transgenic lines in *C. elegans*, which frequently results in the formation of a multicopy DNA array with high expression levels. Therefore, to compare transgenic lines expressing different TOM-1A domain constructs, we first assessed the relationship between TOM-1A expression levels and the synaptic response at the NMJ. TOM-1A mRNA levels were quantified by real-time PCR (qRT-PCR), normalized to *tom-1 (nu468)* and plotted against evoked charge integrals (Fig. 1). As expected, a ∼6 fold increase in TOM-1A mRNA levels greatly reduced the charge integral (72%) relative to *tom-1*(*nu468*). A further increase in TOM-1A mRNA levels to ∼11 fold produced a similar reduction of the evoked response (80%), suggesting that the extent of inhibition was maximal within this range of over-expression (>6 fold).

### Neither TOM-1A SNARE nor ΔSNARE are sufficient for TOM-1A synaptic function

**Figure 2:**
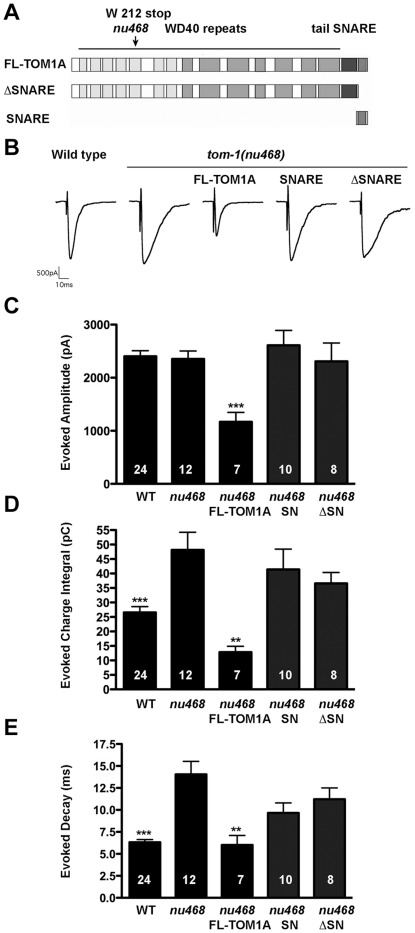
SNARE and ΔSNARE domains of TOM-1A fail to rescue *tom-1(nu468)* mutants. **A.** Schematic showing full-length TOM-1A (SY1242) and the SNARE (SY1230) and ΔSNARE (SY1231) truncated constructs used to generate the integrated transgenics. The position of the early stop at amino acid W212 for *tom-1(nu46*8) is indicated by the arrow **B.** Representative traces of evoked post-synaptic responses and plots of evoked amplitude (***, p = 0.006) (**C**), evoked charge integral (**,p = 0.0014, ***, p = 0.007) (**D**) and evoked half-time decay ((**,p = 0.001, ***, p<0.0001) (**E**). All data are expressed as mean ± SEM. The Mann Whitney T-test was used to determine significance values relative to *tom-1(nu468)*. The sample size (n) is indicated as a number in each bar.

To assess the ability of the SNARE and ΔSNARE domains to rescue the *tom-1* mutant phenotype, we created integrated transgenic lines of either SNARE or ΔSNARE truncated TOM-1A constructs in the *tom-1(nu468)* mutant background, expressed under the cholinergic motor neuron promoter, *Punc-17* (Fig. 2A). By qRT-PCR, TOM-1A SNARE was expressed 23 -fold higher than *tom-1 (nu468)*, and TOM-1A ΔSNARE at ∼17 fold. For full-length TOM-1A, these mRNA levels would be expected to produce maximal synaptic inhibition (Table 1). We then measured cholinergic evoked responses from the NMJs of each of the transgenic lines (Fig. 2B). Unlike full-length TOM-1A over-expression, neither TOM-1A SNARE nor TOM-1A ΔSNARE over-expression significantly reduced the evoked amplitude relative to *tom-1(nu468)* (Fig. 2C). Similarly, the enhanced charge integral and the decay kinetics of the *tom-1(nu648)* mutants were not significantly rescued by over-expression of either TOM-1A SNARE or TOM-1A ΔSNARE, unlike full-length TOM-1A (Fig. 2D,E).

**Table 1:** ΔΔCt-values for TOM-1A transgenic lines.

Strain	Transgenic Line	ΔΔC(t)-values for TOM-1A primers	ΔΔC(t)-values for SNARE primers
**SY1230**	**t** ***om-1*** **; p** ***17*** **:SNARE**	**0.72**	**22.6**
**SY1232**	**t** ***om-1*** **;p** ***17*** **:SNARE-FLAG**	**0.58**	**98**
**SY1231**	**t** ***om-1*** **; p** ***17*** **:**Δ**SNARE**	**16.56**	**1.89**
**SY1233**	**t** ***om-1*** **; p** ***17*** **:**Δ**SNARE-FLAG**	**14.52**	**0.59**
**SY1242**	**t** ***om-1*** **; p** ***17*** **:TOM-1A**	**5.03**	**7.31**
**SY1229**	**t** ***om-1*** **; p** ***17*** **:TOM-1A**	**14.03**	**10.85**

Transgene mRNA levels were determined by qRT-PCR using primers specific for TOM-1A N-terminal (starting at bp1840) and the SNARE domain in the *tom-1(nu468)* mutant background. ΔΔC(t) values were normalized to *tom-1(nu468)* using *act-1* transcript levels as a calibrator.

The linker between the tomosyn N terminus and the SNARE domain has been postulated to act as an intramolecular switch, its interaction with the N terminus freeing the C terminus to inhibit SNARE complex formation [18]. To test the possibility that the linker interaction with the TOM-1A N terminus prevents the N terminus from inhibiting release, we examined evoked release in *tom-1(nu468)* mutants expressing a truncated TOM-1A N-terminal construct cleaved at amino acid 989, which removes both the linker and the SNARE domain (TOM-1A(1-989). Evoked responses from integrants expressing TOM-1A(1-989) also failed to rescue the increased charge integral of *tom-1(nu468)* mutants (charge integral of TOM-1A(1-989) 40.7±3.1 pC, n = 4 Vs 48.2±6.1 pC, n = 12 for *tom-1(nu468)*, p = 0.86), remaining significantly enhanced relative to the wild type (26.5±2.0 pC, n = 12, p = 0.017, data not shown). This result suggests that the failure of TOM-1A ΔSNARE to inhibit release was not due to interactions with the downstream linker

### Both the TOM-1A SNARE and ΔSNARE truncated constructs are stably expressed and localized to nerve cord synapses

**Figure 3:**
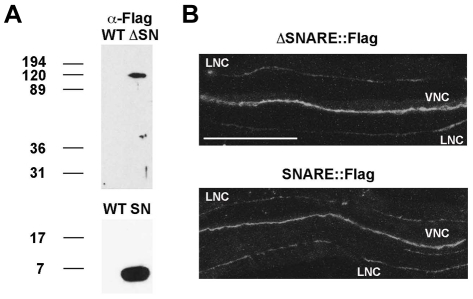
Both TOM1-A SNARE and ΔSNARE are stably expressed and localized at synapses. **A.** The FLAG tagged SNARE and ΔSNARE constructs are of the predicted size on Westerns. **B.** Representative confocal images of SNARE::FLAG and ΔSNARE::FLAG expression in the ventral nerve cord (VNC) anterior to the vulva, the region used for electrophysiological recording. Staining in the lateral nerve cord (LNC) was also observed. Scale bar is 50 µm.

**Figure 4:**
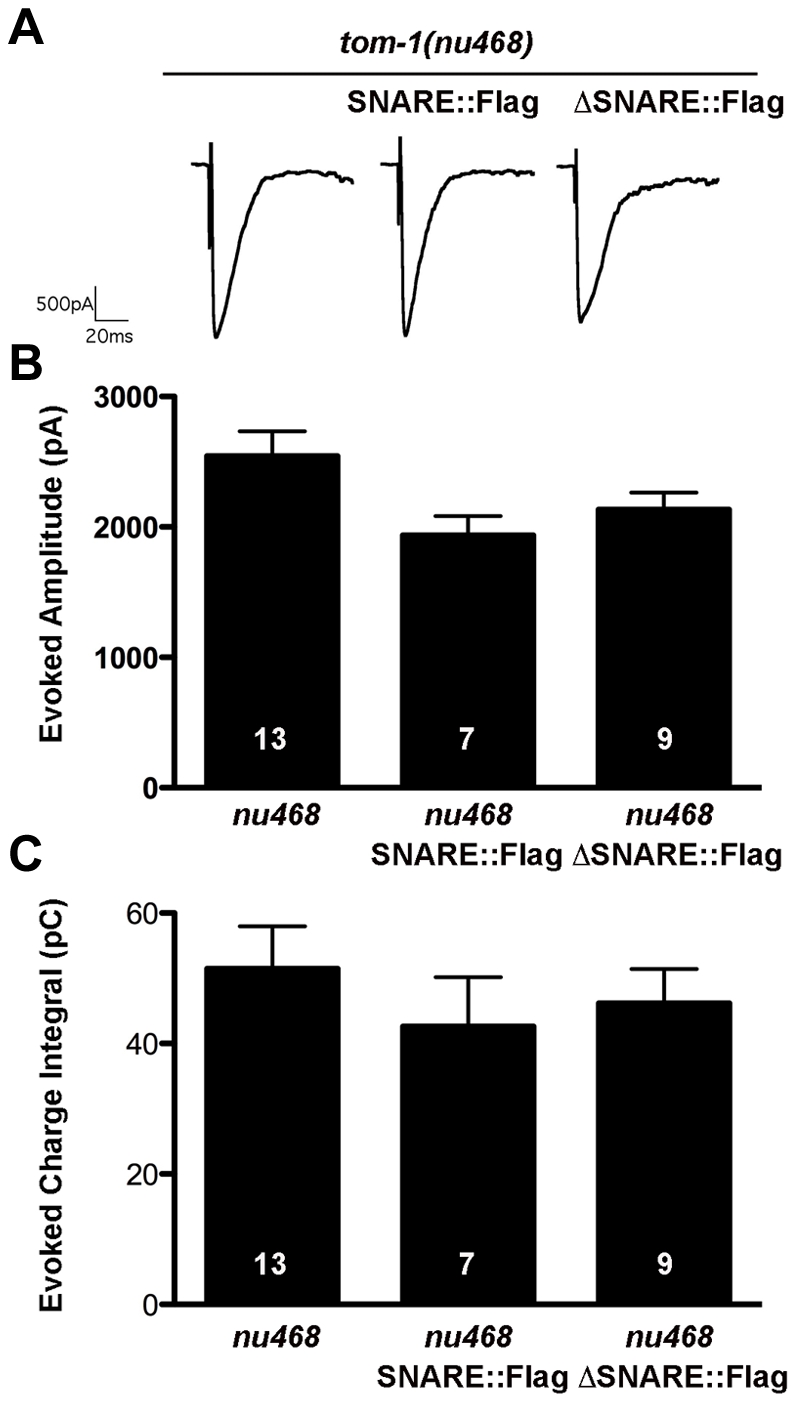
Flag-tagged TOM-1A SNARE and ΔSNARE transgenics phenocopy untagged lines. **A.** Representative evoked response traces for SNARE::FLAG (SY1232) and ΔSNARE::FLAG (SY1233) expressing lines. **B.** Plots of average evoked amplitude and (**C**) evoked charge integral. All data are expressed as mean ± SEM, the sample size (n) is indicated as a number in each bar. Mann Whitney T-tests showed values were not significantly different.

To determine whether the lack of rescue by TOM-1A SNARE and ΔSNARE constructs was due to either poor expression or mislocalization, we generated C-terminal FLAG-tagged versions of both the SNARE and ΔSNARE constructs. The mRNA levels of the FLAG-tagged constructs determined by quantitative RT-PCR were ∼26 fold for SNARE:FLAG and ∼8-fold for ΔSNARE:FLAG relative to *tom-1 (nu468)* (Table 1). Protein extraction and Western blotting of the transgenic worms expressing FLAG-tagged SNARE or ΔSNARE constructs confirmed that proteins of the predicted size (∼7 kDa and ∼110 kDa respectively) were generated (Fig. 3A). We next examined the subcellular localization of the truncated TOM1-A proteins in the cholinergic neurons of *C. elegans* by immunostaining with anti-FLAG antibodies. Staining was imaged along the ventral nerve cord anterior to the vulva, where all electrophysiological recordings were performed (Fig. 3B). The expression pattern of both SNARE:FLAG and ΔSNARE:FLAG was diffusely distributed along the nerve cord in keeping with previous observations of full-length TOM-1A tagged with GFP [11]. Despite their normal expression pattern, both SNARE:FLAG and ΔSNARE:FLAG failed to rescue the *tom-1(nu468)* phenotype, recapitulating the results observed in the untagged lines (Fig. 4). These data indicate that the inability of either the TOM1-A SNARE or ΔSNARE domain to restore TOM-1 function was not due to misexpression.

### Over-expression of TOM-1A SNARE or ΔSNARE fails to inhibit synaptic release in wild-type worms

**Figure 5:**
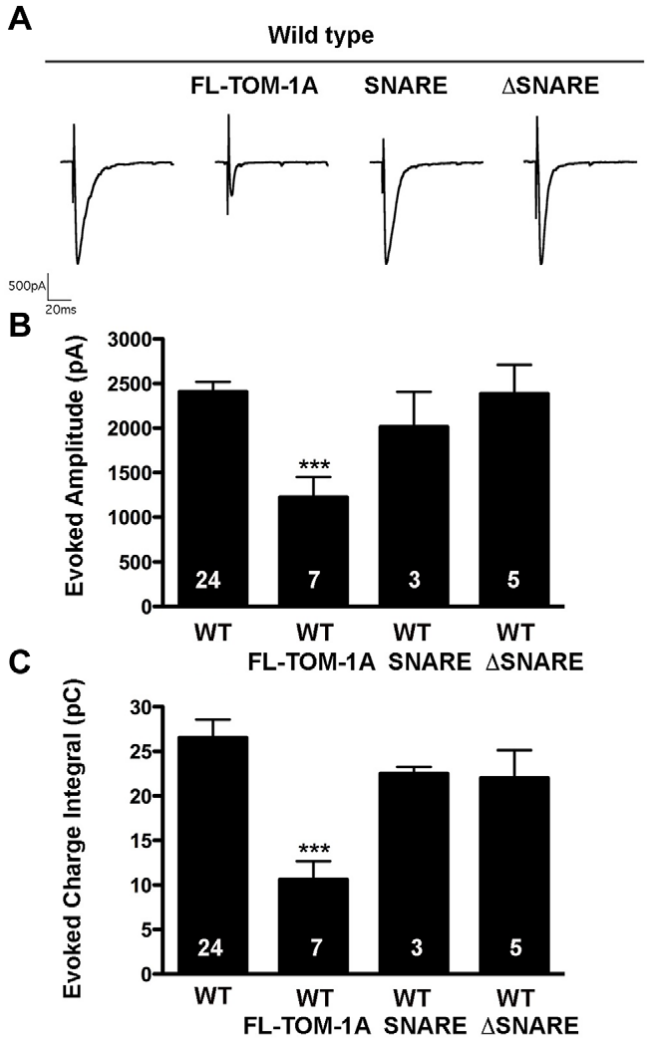
Over-expression of TOM1-A SNARE or ΔSNARE constructs do not inhibit synaptic release in the wild-type background. **A.** Representative evoked traces for full-length TOM-1A (SY1237), SNARE (SY1234) and ΔSNARE (SY1235) expressing transgenes in the wild-type background. (**B**) Average evoked amplitude and (**C**) Average charge integral were only significantly reduced by full-length TOM-1A relative to wild type (***, p = 0.0005, and p = 0.0007 for B and C, respectively). All data are expressed as mean ± SEM, the sample size (n) is indicated as a number in each bar, significance values obtained with the Mann Whitney T-test.

In cultured neurons, in which endogenous tomosyn is present, expression of either tomosyn SNARE or tomosyn ΔSNARE has been reported to inhibit synaptic transmission [12], [18]. To address whether *C. elegans* TOM-1A SNARE or ΔSNARE expression has an inhibitory effect in the presence of endogenous TOM-1, we crossed the truncated lines into the wild-type background. Evoked synaptic responses were not inhibited by either TOM-1A SNARE or ΔSNARE, whereas full-length TOM-1A over-expression caused a significant decrease in evoked charge integral (Fig. 5).

### Co-expression of SNARE and ΔSNARE constructs fails to reconstitute TOM-1A function

**Figure 6:**
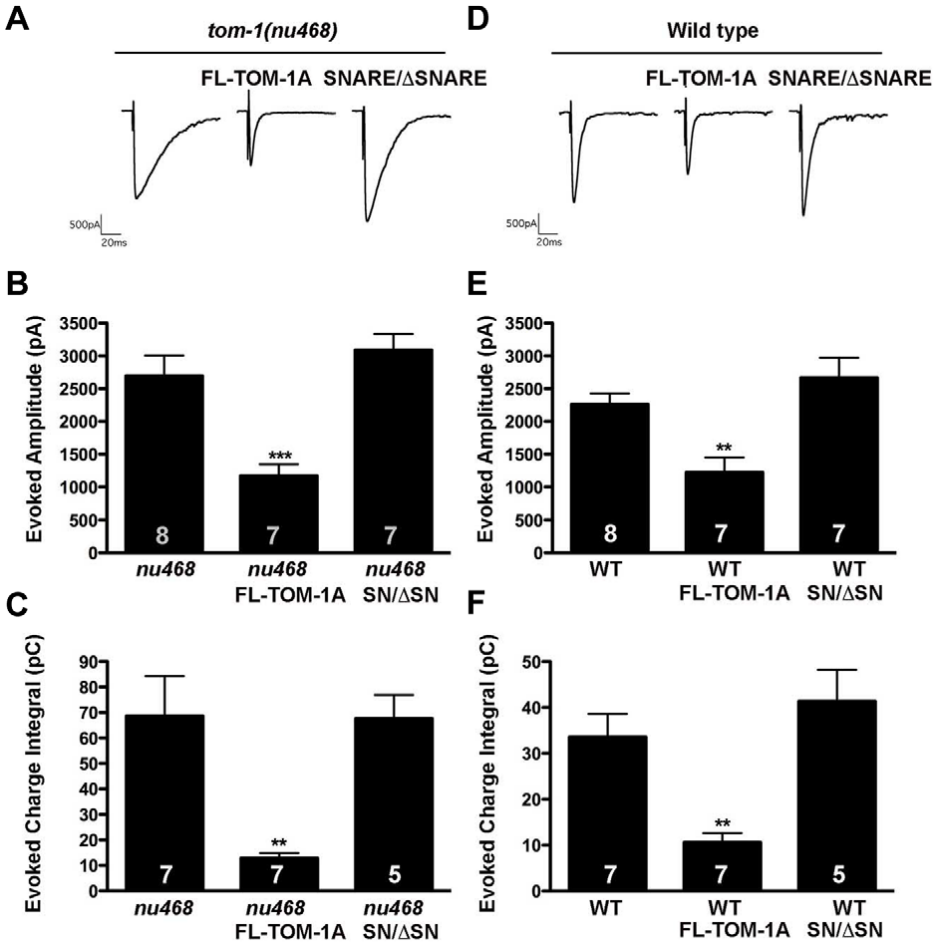
Co-expression of SNARE and ΔSNARE constructs failed to reconstitute TOM-1A function. **A.** Representative evoked response traces for *tom-1(nu468)*, and with full-length TOM-1A over-expression (SY1242) or co-expression of SNARE and ΔSNARE (SY1239), (**B**) average evoked amplitudes (***, p = 0.0006) and (**C**) average evoked charge integrals (**, p = 0.0021). (**D**) Representative evoked response traces for wild type alone, and with TOM-1A over-expression (SY1237) or co-expression of SNARE and ΔSNARE (SY1240), (**E**) average evoked amplitudes (**, p = 0.0033) and (**F**) evoked charge integrals (**, p = 0.0037). All data are expressed as mean ± SEM, the sample size (n) is indicated as a number in each bar, significance values obtained with the Mann Whitney T-test.

To address whether co-expression of the TOM-1A SNARE and TOM-1A ΔSNARE constructs could reconstitute TOM-1A function, we co-expressed both constructs (SNARE/ΔSNARE) in the *tom-1(nu468)* background. Unlike full-length TOM-1A, co-expression of TOM-1A SNARE/ΔSNARE failed to rescue the enhanced evoked response of the *tom-1(nu468)* mutant (Fig. 6A–C). Similarly, co-expression of TOM-1A SNARE/ΔSNARE failed to recapitulate the inhibitory effect of full-length TOM-1A over-expression in the wild-type background (Fig. 6 D–F).

## Discussion

Although the inhibitory role of tomosyn in exocytosis is well established, the molecular events underlying this negative regulation remain to be fully elucidated [21]. To further our understanding of tomosyn function, we examined the inhibitory capacity of the two conserved tomosyn domains *in vivo*. Our results indicate that the integrity of *C. elegans* TOM-1 is critical for its inhibitory function, as neither TOM-1A SNARE nor ΔSNARE were able to restore TOM-1 function, when expressed separately or together in *tom-1* mutants.

Although biochemical evidence strongly implicates the tomosyn SNARE domain in the regulation of SNARE complex formation [6], [12], [16], the inhibitory capacity of this domain depends on the experimental context [6], [17], [18]. Whereas the tomosyn SNARE domain expressed in cultured SCG neurons [12] or applied to inverted PC12 cell plasma membrane sheets [6] inhibits secretion, evoked release is unaffected in both chromaffin cells [17] and, as shown here, in *C. elegans* motor neurons. What experimental variable might account for these different outcomes? Since tomosyn SNARE-dependent synaptic inhibition in SGC cells has only been assayed in wild-type neurons, it is possible that the SNARE domain may inhibit release by altering intramolecular or intermolecular interactions of endogenous tomosyn. However, the inability of the tomosyn SNARE domain to inhibit release when endogenous tomosyn was present in wild type *C. elegans* as well as chromaffin cells argues against this possibility. Alternatively, the ability of the SNARE domain to impact SCG neuron and PC12 ghost cell secretion may reflect the achievement of higher expression levels by microinjection or direct application in these cells. While we cannot rule out this explanation, the lack of an inhibitory effect of SNARE over-expression in either chromaffin cells or *C. elegans* neurons, at levels effective for full-length tomosyn, argues against the physiological relevance of the observed inhibition in SCG and PC12 ghost cells. It is also possible that the molecular events underlying the inhibitory capacity of the tomosyn SNARE domain are differentially tuned in the neurons of vertebrates and *C. elegans*. The vertebrate tomosyn SNARE domain has been shown to readily form tomosyn SNARE complexes in cell free assays and to compete with synaptobrevin in the assembly of SNARE complexes [6], [16]. We know from *in vitro* assays that the *C. elegans* TOM-1 SNARE domain is much less efficient than the SNARE domain of C. *elegans* synaptobrevin (SNB-1) in promoting the assembly of recombinant SNARE complexes [10]. Moreover, the *C. elegans* SNAP-25 homolog (RIC-4) is much less efficient than vertebrate SNAP-25 in facilitating both TOM-1 and SNB-1-containing SNARE complex formation *in vitro*. Substituting vertebrate SNAP-25 for RIC-4 greatly enhances levels of both *C. elegans* TOM-1 and SNB-1 containing SNARE complexes *in vitro* [10]. Thus, we postulate that *in vivo*, *C. elegans* TOM-1 SNARE may be much less effective in inhibiting fusogenic SNARE complex formation relative to the vertebrate tomosyn SNARE domain in SCG cells. For this explanation to fit the current data, it would suggest that there must also be differences in the ability of the vertebrate tomosyn SNARE domain to impact dense core granule secretion in chromaffin cells relative to SCG synapses.

In contrast to the SNARE domain, expression of the tomosyn N-terminal domain inhibits release from both chromaffin [17] and SCG cells [12], [18], [20], as does full-length tomosyn lacking SNARE-syntaxin interactions in PC12 cells [19]. Yet, TOM-1 lacking the SNARE domain fails to rescue the *C. elegans tom-1* mutant phenotype. A recent analysis of rat tomosyn mutants indicates that tomosyn promotes the formation of SNARE complex oligomers, providing a possible second mechanism by which tomosyn may limit the assembly of fusogenic SNARE complexes through the sequestration of SNARE proteins [12]. Although the precise mechanism underlying SNARE complex oligomerization is unknown, the tomosyn N-terminal domain recapitulates this effect in cell free assays [12]. Since the isolation of native SNARE complexes from *C. elegans* has yet to be achieved, we are unable to address whether *tom-1* mutants show a similar reduction in SNARE complex oligomerization. Regardless, the inability of TOM-1A ΔSNARE over-expression to rescue *tom-1* mutants or inhibit release in wild type *C. elegans* suggests that, in this *in vivo* context, expression of full-length TOM-1 is required for functionality.

How might linkage of the two TOM-1 domains within the full-length protein contribute to the ability of TOM-1 to negatively regulate synaptic transmission? Recently, evidence for a third tomosyn inhibitory mechanism has emerged, which involves a calcium-dependent interaction between the rat tomosyn N terminus and the vesicle-associated calcium-sensor, synaptotagmin [20]. The binding of tomosyn to synaptotagmin interferes with the *in vitro* membrane-bending ability of synaptotagmin, a function implicated in the vesicle fusion process [22], [23]. Furthermore, injection of the synaptotagmin cytoplasmic domain represses the ability of the tomosyn N-terminal domain to inhibit release from cultured SCG neurons, suggesting the interaction between the tomosyn N-terminal domain and endogenous synaptotagmin underlies this inhibitory effect. Interestingly, synaptotagmin binding to full-length tomosyn also enhances the ability of the tomosyn SNARE domain to form tomosyn SNARE complexes [20]. These data imply that the tomosyn N-terminal interaction with synaptotagmin may favorably position the C-terminal tomosyn SNARE domain to initiate tomosyn SNARE complex assembly. This result suggests that the integrity of tomosyn could facilitate simultaneous interference with synaptotagmin function and SNARE complex assembly, via the linked N- and C-terminal domains, respectively. In this model, the spatial proximity of the two tomosyn domains, would be an important requirement for the dual inhibition and may explain why the integrity of TOM-1 is essential for inhibitory function at *C. elegans* synapses.

In conclusion, we have conducted the first *in vivo* analysis of TOM-1 structure-function in a *tom-1* mutant background. Based on our results we conclude that the physical link between the N- and C-terminal domains is critically important for the normal function of TOM-1. This result differs from previous studies in cultured mammalian cells, in which over-expression of the SNARE domain produced variable results and the N terminus inhibited secretion. It remains to be seen whether expression of either the tomosyn SNARE or delta-SNARE domains in the recently available mouse tomosyn mutants restores tomosyn function *in vivo* [18].

## Materials and Methods

### Genetics

Nematode strains were maintained at 20–25°C on standard NGM media plates seeded with OP50 bacteria. The wild type used was Bristol N2 and the *tom-1* mutant *KP3293, tom-1(nu468)*. TOM-1 constructs were SY1229, *tom-1(nu468)*;*jaIs1078*[*Punc17*:*tom-1A*(+);*Pmyo-2:GFP*]; SY1242, *tom-1(nu468)*;*jaIs1052*[*Punc17*:*tom-1A*(+);*Pmyo-2:GFP*]; SY1230, *tom-1(nu468)*;*jaIs1079*[*Punc17:tom-1A SNARE*;*Pttx:RFP*]; SY1231, *tom-1(nu468)*; *jaIs1080*[*Punc17*:*tom-1A* ΔSNARE;*Pttx:RFP*]; SY1513, *tom-1(nu468);jaI1098*[*Punc17;tom-1A TOM1A(1-989);Pttx:RFP*]; SY1232, *tom-1(nu468)*;*jaIs1081*[*Punc17:tom-1A SNARE:FLAG;pmyo-3:GFP*]; SY1233, *tom-1(nu468)*;*jaIs1082* [*Punc17: tom-1A* Δ*SNARE:FLAG;pttx-3:GFP*]; SY1234, N2;*jaIs1079*; SY1235, N2;*jaIs1080*; SY1237, N2;jaIs1052; SY1239, *tom-1(nu468)*;*jaIs1079*;*jaIs1080*; SY1240, N2;*jaIs1079*;*jaIs1080*.

Crosses were performed using standard genetics techniques, and the presence of the *tom-1(nu468)* mutation was confirmed by sequencing.

### Tomosyn constructs and transgenes

#### 1) Full-length *tom-1A*

Full-length *tom-1A* cDNA was amplified from *jaIs1052* strain [10] using the primers **GTAGCATGCGCTGGGGTATTGCAAAAAGAG** and **GTCGCATGCCTAGAAGTTGTACCACTTC** and TOPO-cloned, creating pAB29. pUNC-17::TOM-1A from pAB29 was cloned into pAB30 using SphI restriction sites and the resulting plasmid was named pAB32.

#### 2) TOM-1A SNARE

The *tom-1A* SNARE domain (aa 1059–1124) was amplified from pAB29 using primers **GCGGATCCATGCAAATGGATAGAGCACAAGC** and **CGTGGCCACTAGAAGTTGTACCACTTC** and TOPO-cloned, creating pAB37. The *tom-1A* SNARE domain was then subcloned into pAB36 containing *pUNC-17* using BamHI/MscI restriction sites, creating pAB40.

#### 3) TOM-1A ΔSNARE

The *tom-1A* Δ*SNARE* domain (aa 1–1045 ) includes the WD40 repeats and the downstream 57 amino acid linker, based on conserved sequence alignments [24] amplified from pAB29 using primers **AGAGTCATCCCTCAGAACAG** and **GTCTAGGATCCATGCATCGGATTCACTCCAGAACTATTC**, TOPO-cloned creating pAB47. *tom-1A* Δ*SNARE* was subcloned into pAB36 containing *pUNC-17* using BamH restriction sites, creating pAB48.

#### 4) TOM-1A (1-989) lacking the linker and SNARE domain

TOM-1A(1-989) was amplified from pAB29 using primers **AGAGTCATCCCTCAGAACAG** and **TATGCGGCCGCTGACTCGCCTGTTTGCTCGGCAATTTC** and topo-cloned, creating pAB46. The *tom-1A* Δ*linker* was subcloned into pAB36 containing pUNC17 using BamHI/NotI restriction sites, creating pAB49.

#### 5) TOM-1A SNARE:FLAG

The NsiI restriction site (underlined) was introduced by site-directed mutagenesis using primers **GTGGTACAACTTCATGCATTAGTGGCCAAAGGAC** and **GTCCTTTGGCCACTAATGCATGAAGTTGTACCAC** using pAB40 as a template, creating pAB43. The FLAG oligos with NsiI sticky ends and 5′-phosphorylated were made as separate oligonucleotides, **PTGATTACAAGGATGACGACGATAAGCTTATGCA** and **TAAGCTTATCGTCGTCATCCTTGTAATCATGCA **, annealed and ligated into the NsiI site of pAB43, creating pAB50.

#### 6) TOM-1A ΔSNARE:FLAG

The NsiI restriction site was included in the primer **GTCTAGGATCCATGCATCGGATTCACTCCAGAACTATTC** to bypass the site-directed mutagenesis step. Annealed FLAG oligonucleotides were ligated into pAB48 NsiI restriction site, creating pAB58.

### Real-time PCR

Quantitative real-time PCR was performed as described previously [10]. Briefly, *C. elegans* total RNA was isolated using a Trizol reagent as described by the manufacturer (Invitrogen, Carlsbad, California, United States). mRNA was reverse transcribed using the SuperScript III First Strand Synthesis Kit with oligo-dT primers (Invitrogen Carlsbad, California, United States). Real-time PCR was preformed using the following target specific primers: for *tom-1A* N-terminal (Forward-TCATCGTACGGTATCATTGC and Reverse- AGCTTCCAGACTGATTGGAG) which targeted exon 12, for the SNARE domain (Forward-GCCATGGCTTTACAGAACTT and Reverse- TCTCGAGGATAAACTCATTGC) which targeted exon22/23, and for *act-1* (Forward-GCTGGACGTGATCTTACTGATTACC and Reverse-GTAGCAGAGCTTCTCCTTGATGTC). SYBR green (Biorad) was used for amplicon detection and quantitation using the MJ Research Opticon2 real-time thermocycler (Bio-Rad, Hercules, California, United States). Relative mRNA levels were quantified using the method detailed by [25]. Actin was used as a reference for calibration [26]. The levels for both *tom-1* and the SNARE domain are reported as the fold difference relative to the calibrator, *tom-1 (nu468)*.

### Biochemistry

#### 1) Liquid Culture

L1 stage worms were harvested from 6 freshly-starved 100 mm agarose plates and added, along with concentrated HB101 bacteria, to 500 ml S medium supplemented with 5 ml 10,000 U/ml penicillin (Cellgro), 10 mg/ml streptomycin (Cellgro) and 10,000 U/ml nystatin (Sigma) in a 2.8 L fernbach flask. After 3 days of growth at 20°C, adult worms were harvested through a 35 µM nitex filter. Worms left in the filter were washed once with M9, 1X lysis buffer (50 mM HEPES pH 7.4, 1 mM EGTA, 1 mM MgCl_2_, 100 mM KCl, 10% glycerol, 0.05% NP-40) and 1X lysis buffer containing a complete Mini, EDTA-free protease inhibitor cocktail tablet (Roche; 1 tablet/12 mls). Worms were spun down at 800 g for 2 minutes between washes. A 1∶1 mix of worms∶lysis buffer was slowly pipetted into liquid nitrogen then ground to a fine powder in a mortal and pestle.

#### 2) Extract Preparation

Thawed ground worm powder was sonicated using a Branson sonication tip for 3 minutes (15 seconds on, 45 seconds off) at 30% amplitude and for 30 seconds at 40% amplitude. Samples were cooled in an ice bath for 2 minutes in between each minute of sonication. Sonicated samples were centrifuged in a Sorvall Ultra80 centrifuge, using a TH-641 rotor, for 11.5K RPM for 10 minutes, and then the supernatant was centrifuged at 29K RPM for 20 minutes. The supernatant was frozen in liquid nitrogen in 1 ml aliquots and stored at −80°C.

#### 3) Immunoprecipitation

100 µl of FLAG antibody-conjugated agarose beads (Sigma F2426) were washed twice with 1 ml PBST, once with 1 ml PBS, twice with 1 ml 0.1 M glycine and twice with 1 ml ice cold lysis buffer with 0.1 mM DTT. Beads were centrifuged at 4°C at 4K RPM for 2 minutes between washes. Beads were rotated for 2 hours at 4°C with 1 ml of clarified extract. Beads were briefly rinsed twice then washed three times, by rotating for 5 minutes at 4°C, with 1 ml 0.1 mM DTT lysis buffer. Beads were incubated with 9 µl of 600 µg/ml FLAG peptide at 4°C for 1 hour in Protein LoBind tubes (Epindorf). Supernatant was transferred to fresh tubes and mixed with equal volume 2X Laemmli SB and stored at −20°C.

#### 4) Western blotting

Samples were analyzed by 12% SDS-PAGE (0.1% SDS). The nitrocellulose membrane was blocked at RT in 5% milk/TBST for 1 hour then incubated with HRP-conjugated 1° anti-FLAG antibody (Sigma A8592) at a 1∶500 dilution in TBST overnight at 4°C. Membrane was washed four times for 10 minutes with TBST shaking at RT then incubated with 2 ml HRP substrate (Amersham) for 5 minutes prior to exposure to film.

### Immunohistochemistry

Immunohistochemistry was performed on dissected split-open worms, as previously described [27] after fixation with 4% paraformaldehyde in PBS for 30 minutes. Preps were then washed 3× with TBST for 10 minutes, before blocking with 5% BSA for 1 hour. Mouse antibodies against FLAG (Sigma) were used at a final dilution of 1∶100 in PBS and 0.5% Triton X-100 with 5% BSA overnight. Anti-mouse tetramethylrhodamine isothiocyanate-conjugated secondary antibody (Jackson ImmunoResearch, West Grove, PA) was used at a 1∶500 dilution for 1 hour. Images were obtained with a 60× objective on an Olympus Optical FV- 500 laser-scanning confocal microscope.

### Electrophysiology

Electrophysiological methods were as previously described [27] with the following modifications: Ventral body wall muscle cells were recorded in the whole-cell voltage-clamp mode (holding potential −60 mV) using an EPC-10 patch-clamp amplifier and digitized at 1 kHz. The extracellular solution consisted of (in mM): NaCl 150; KCl 5; CaCl_2_ 5; MgCl_2_ 4, glucose 10; sucrose 5; HEPES 15 (pH 7.4, ∼340mOsm). The patch pipette was filled with (in mM): KCl 120; KOH 20; MgCl_2_ 4; (*N*-tris[Hydroxymethyl] methyl-2-aminoethane-sulfonic acid) 5; CaCl_2_ 0.25; Na^2^ATP 4; sucrose 36; EGTA 5 (pH 7.2, ∼315mOsm). Evoked responses were stimulated with a 2 ms depolarizing pulse delivered via a pipette placed on the anterior ventral nerve cord. Data were acquired using Pulse software (HEKA, Southboro, Massachusetts, US) and subsequently analyzed and graphed using Pulsefit (HEKA), Mini Analysis (Synaptosoft Inc., Decatur, Georgia, US) and Igor Pro (Wavemetrics, Lake Oswego, Oregon, US).
